# The chrondoprotective actions of a natural product are associated with the activation of IGF-1 production by human chondrocytes despite the presence of IL-1β

**DOI:** 10.1186/1472-6882-6-13

**Published:** 2006-04-07

**Authors:** Mark JS Miller, Salahuddin Ahmed, Paul Bobrowski, Tariq M Haqqi

**Affiliations:** 1 Center for Cardiovascular Sciences, Albany Medical College, Albany, New York, USA; 2 Department of Medicine, Division of Rheumatic Diseases, Case Western Reserve University School of Medicine, Cleveland, Ohio, USA; 3 Rainforest Nutritionals, Inc., Raleigh, North Carolina, USA

## Abstract

**Background:**

Cartilage loss is a hallmark of arthritis and follows activation of catabolic processes concomitant with a disruption of anabolic pathways like insulin-like growth factor 1 (IGF-1). We hypothesized that two natural products of South American origin, would limit cartilage degradation by respectively suppressing catabolism and activating local IGF-1 anabolic pathways. One extract, derived from cat's claw (*Uncaria guianensis*, vincaria^®^), is a well-described inhibitor of NF-κB. The other extract, derived from the vegetable *Lepidium meyenii *(RNI 249), possessed an uncertain mechanism of action but with defined ethnomedical applications for fertility and vitality.

**Methods:**

Human cartilage samples were procured from surgical specimens with consent, and were evaluated either as explants or as primary chondrocytes prepared after enzymatic digestion of cartilage matrix. Assessments included IGF-1 gene expression, IGF-1 production (ELISA), cartilage matrix degradation and nitric oxide (NO) production, under basal conditions and in the presence of IL-1β.

**Results:**

RNI 249 enhanced basal IGF-1 mRNA levels in human chondrocytes by 2.7 fold, an effect that was further enhanced to 3.8 fold by co-administration with vincaria. Enhanced basal IGF-1 production by RNI 249 alone and together with vincaria, was confirmed in both explants and in primary chondrocytes (P <0.05). As expected, IL-1β exposure completely silenced IGF-1 production by chondrocytes. However, in the presence of IL-1β both RNI 249 and vincaria protected IGF-1 production in an additive manner (P <0.01) with the combination restoring chondrocyte IGF-1 production to normal levels. Cartilage NO production was dramatically enhanced by IL-1β. Both vincaria and RNI 249 partially attenuated NO production in an additive manner (p < 0.05). IL-1β – induced degradation of cartilage matrix was quantified as glycosaminoglycan release. Individually RNI 249 or vincaria, prevented this catabolic action of IL-1β.

**Conclusion:**

The identification of agents that activate the autocrine production of IGF-1 in cartilage, even in the face of suppressive pro-inflammatory, catabolic cytokines like IL-1β, represents a novel therapeutic approach to cartilage biology. Chondroprotection associated with prevention of the catabolic events and the potential for sustained anabolic activity with this natural product suggests that it holds significant promise in the treatment of debilitating joint diseases.

## Background

Osteoarthritis is a painful and debilitating joint condition that affects hundreds of millions worldwide [1,2]. Despite the prevalence of the disease the current therapeutic options have significant limitations. The popular non-steroidal anti-inflammatory class of therapeutics (NSAIDs), which block cyclo-oxygenase (COX), provide symptomatic relief but do not abrogate the underlying disease process [3]. Indeed, it is well appreciated that cartilage destruction can proceed unabated despite suppression of inflammation [4]. To this dilemma, the COX-2 specific class of NSAIDs has revealed an increase in the risk for cardiovascular and heart disease [5,6], although their design was an attempt to reduce complications associated with non-specific COX inhibitors [7]. Thus the quest to develop new therapeutic entities has taken on greater impetus and yet additional uncertainty.

Patients often supplement pharmaceutical strategies for managing arthritis with complementary medicines. These may include acupuncture [8], nutraceuticals [9-11], and botanicals [12,13]. Glucosamine and chondroitin based products dominates the nutraceutical approach to arthritis. Glucosamine and chondroitin are structural elements of cartilage and matrix and their proposed therapeutic benefits center on the assumption that ingestion of these matrix elements, despite their poor absorption [14,15], will replace matrix that is lost as a result of the catabolic process. Not surprisingly large amounts are required and the onset of action with this approach is on the order of months [16-19]. The recently published GAIT study [20] indicated that glucosamine, chondroitin as individual treatments or in combination failed to demonstrate significant differences from placebo in the relief of mild to moderate osteoarthritis of the knee. This observation may reflect that these are structural elements only and do not directly modify the processes of joint destruction or repair.

On the other hand botanicals, especially those with redox-based actions, hold promise in the treatment of chronic inflammation [21-26]. Green tea catechins, especially epigallocatechin gallate (EGCG), have been shown to limit human cartilage degradation in vitro [20,26] and maintain joint architecture in the collagen-induced arthritis (CIA) animal model [26]. This anti-inflammatory action is thought to be the result of inhibition of transcriptional events, particularly prevention of NF-κB activation by cytokines and oxidants. NF-κB is a critical transcription factor in chronic inflammation and is a desirable target for new therapeutics, including pharmaceutical development, as it regulates numerous genes that contribute to the inflammatory process [28-30]. Of particular note for joint dysfunction, NF-κB regulates the production of matrix metalloproteases (MMPs) by chondrocytes [31]. During inflammation or injury chondrocytes release MMPs, which in turn degrade the cartilage matrix, releasing glycosaminoglycans (GAG), which can then be readily quantified as an index of catabolic events in cartilage. Indeed, these events were reproduced in this study with human knee cartilage explants where IL-1β treatment causes GAG release from explants.

While the developmental pipeline for new therapeutics has focused on symptomatic relief (NSAIDs) and approaches to alleviate joint destruction (MMP suppression), there are few approaches that also address joint restoration or activation of anabolic, repair pathways. It has been well appreciated that inflammation and infection silences the expression of repair pathways like IGF-1 [32,33]. Concomitant with a suppression of local, autocrine production of IGF-1 during inflammation is an uncoupling of the signal transduction pathways for exogenous Growth Hormone (GH) and IGF-1 [34-36]. This combination of lost tissue responsiveness to circulating levels of anabolic factors and compromised local production results in a sustained loss of anabolic tone. For joints, this may include supporting musculature as well as cartilage and bone [37]. This problem is more apparent for the elderly where these events are superimposed on an age-related loss in IGF-1 activity. For these reasons, Cappola et al [38] observed that those elderly individuals with a combination of high IL-6 levels and low IGF-1 levels are at the greatest risk for enhanced morbidity and mortality.

These factors also relate to the increasing incidence of osteoarthritis and joint dysfunction with age [39] and the reduced growth associated with pediatric chronic inflammatory bowel disease, where anti-IL-6 antibodies restored linear growth and IGF-1 levels independent of nutrition [40]. Indeed, there appears to be excellent evidence that growth restriction with chronic inflammation, including arthritis, is the result of suppression of IGF-1 production and activity, independent of GH, and in response to IL-6 [41,42]. Supplemental IGF-1 restored linear growth in these models, linking the suppression of IGF-1 to compromised anabolic activity [43].

In the present study we hypothesized that a combination of a cat's claw (*Uncaria guianensis*) extract devoid of immunostimulatory oxindole alkaloids [44] and an extract of the Andean vegetable *Lepidium meyenii*, may provide the desired therapeutic innovation for compromised anabolic tone. The rationale for this hypothesis centered on our previous work with *Uncaria guianensis *extract vincaria, as a potent inhibitor of NF-κB, TNFα and associated anti-inflammatory and cytoprotective actions [25,44-46]. Additionally, vincaria has successfully completed a small trial for osteoarthritis of the knee demonstrating a rapid reduction in pain and improved function [47].

*Lepidium meyenii *extracts had previously been demonstrated to have pro-fertility actions [48-51], an application that is not intuitive for treating arthritis. The pro-fertility actions of *Lepidium meyenii *could not be explained on the basis of an augmentation of sex steroid or gonadotrophin pathways [48,49] and the mechanism underlying these benefits has remained elusive until now. However, based on this ethnomedical knowledge and our own observations that these extracts were anabolic in farmed fish (unpublished reports) whose growth is critically dependent on IGF-1, we hypothesized that the central mechanism of action for *Lepidium meyenii *extracts was the autocrine activation of IGF-1. If our hypothesis was to be validated then this would rapidly open a new approach to the management of arthritis.

The present study was designed to address these issues with a focus on the functional consequences of maintaining IGF-1 gene activity and production in human cartilage explants. The study had goals of determining if RNI 249 could activate human chondrocyte IGF-1 gene expression and maintain IGF-1 production in the face of pro-inflammatory signals, IL-1β, that normally silence the IGF-1 gene.

## Methods

### Reagents

Tissue culture medium and related reagents were purchased from either Mediatech (Herndon, VA) or InVitrogen (Carlsbad, CA). Recombinant human IL-1β was purchased from R&D Systems (St Paul, MN), and other chemicals were purchased from Sigma-Aldrich (Saint Louis, MO). The extracts, vincaria (RN180) or RNI 249, and their combination (Reparagen™) were supplied by Rainforest Nutritionals, Inc and were dissolved in water and filtered through a 0.45 μm filter under vacuum prior to use. Vincaria is an alkaloid depleted water based extract of *Uncaria guianensis *that is standardized for antioxidant activity (DPPH radical quenching) and an oxindole alkaloid content of less than 0.1 mg/g as determined by HPLC. RNI 249 consists of a polar extract of *Lepidium meyenii *that is standardized for its DPPH quenching activity prior to combining with an inert stabilizing material.

### Human chondrocytes culture and cartilage explants

Human OA cartilage samples were procured through the Tissue Procurement Facility of University Hospitals of Cleveland/Case Western Reserve University and with prior approval of the Institutional Review Board of University Hospitals of Cleveland. Cartilage samples were obtained from a total of 10 subjects, 6 females and 4 males, representing several racial groups (Hispanic, African-American and Caucasian, with a mean age of 62 yrs). The cartilage samples were obtained from patients undergoing total arthroplasty of the knee due to degenerative joint diseases. In all cases care was taken to use only "macroscopically normal" cartilage samples. For replication of experiments chondrocytes from age and sex matched cartilage samples were used. No samples were exposed to radiation solely for the purpose of these studies but almost all the patients will have received X-rays as part of their clinical presentation during the execution of care. The same donor tissue was not used in all experiments but untreated controls were included in all protocols.

Chondrocytes were prepared by the enzymatic digestion of knee cartilage as previously described [20,23,26]. Chondrocytes were plated (1 × 10^6 ^cells/ml) in 35 mm culture dishes (Becton-Dickinson, Mountain View, CA, USA) and cultured in DMEM:F12 (Mediatech, Herndon, VA) supplemented with 10% FCS and 1% Penn:Strep for 72 hrs at 37^°^C and 5% CO_2 _in a tissue culture incubator. Chondrocytes were serum-starved overnight and then exposed to RNI 249 (50 μg/ml), vincaria (10 μg/ml) alone or in combination in fresh serum-free medium for 1 hr prior to the addition of IL-1β. Cell viability before plating was monitored by the MTT assay (Cell Viability and Proliferation Assay) according to the instructions of the manufacturer (R&D Systems). In some cases, chondrocyte viability after exposure to the different agents was determined by Trypan blue exclusion assay.

Full-thickness cartilage slices (20–25 mg) were dissected from the cartilage using sterile scalpel blade (Feather Safety Razor Co., Japan). Four to five cartilage pieces (approximately equal in size and weight) were transferred to each well of a 24-well, flat bottom plate (Nunc, Denmark) containing DMEM:F-12 (1:1) supplemented with antibiotics and 10% FCS and cultured for 24 hours. Subsequently the cartilage explants were cultured overnight in serum free media. The cartilage explants were treated with IL-1β alone or with IL-1β + vincaria or RNI 249, alone or in combination for 72 hrs in serum free media. Explants cultured in the absence of IL-1β, vincaria or RNI 249, were used as controls. Additionally, the actions of RNI 249 and vincaria singularly and together were examined independently of IL-1β exposure (5 ng/ml). Where appropriate, explants were exposed to vincaria or RNI 249 were 15 minutes prior to the treatment with IL-1β. Total glycosaminoglycan present in the culture supernatant was estimated as described below. The doses of RNI 249 and vincaria that were evaluated were the lowest doses demonstrating effectiveness. However full dose-response curves were not performed as the focus was on potential additive effects and dosing that is likely to be exposed in the clinical setting.

### Quantitative RT-PCR

Total cytoplasmic RNA was prepared from primary cultures of human chondrocytes using a commercially available kit according to the instructions of the manufacturer (Qiagen, Valencia, CA). Real time quantitative RT-PCR with internal fluorescent hybridization probes was performed as previously described [22] and the IGF-1 gene expression was quantified using a commercially available Gene Expression Assay kit (Applied Biosystems, CA). Expression of IGF-1 mRNA was normalized to β – actin mRNA expression, and the results were expressed as fold induction relative to controls.

### IGF-1 ELISA

Human IGF-1 level in chondrocyte culture or cartilage explant media was quantified using a commercially available Human IGF-1 ELISA kit (R & D Systems) per manufacturer's directions.

### Quantitation of glycosaminoglycans

At the end of culture period, the culture medium was collected from each group. A 50 μl aliquot of the collected supernatant from each sample was utilized to estimate the total glycosaminoglycan (GAG) concentration by a colorimetric method employing DMMB as previously described [53]. Color intensity was read spectrophotometrically at 535 nm using the Lambda 25 spectrophotometer (Perkin-Elmer, CT) and the values were derived from a standard curve prepared using different concentrations of chondroitin sulfate. Results are expressed as micrograms of glycosaminoglycan released per milligram of cartilage tissue.

### Nitric oxide production

Levels of nitrate/nitrite were measured in cartilage explant and chondrocyte culture media by using the Griess reaction (colorimetric assay) after conversion of the nitrate to nitrite using a commercially available kit (Molecular Probes, OR).

### Statistical analysis

Cartilage samples from donors were included in control and test conditions, in other words the influence of subject variability was negated by including explants or cultures from individual donors in both the control and test samples. Data was analyzed using the software package, Instat^®^, and the analysis included one ay ANOVA followed by an appropriate post hoc test (Dunnett's). Values are expressed as mean ± SEM. Differences were considered significant at P <0.05.

## Results

### IGF-1 expression and production by human cartilage

**Figure 1:**
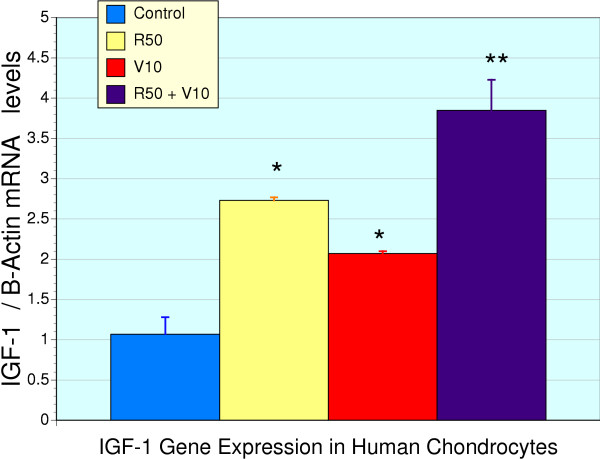
**Enhanced expression of IGF-1 gene in human chondrocytes**. IGF-1 gene expression referenced by the activity of β – actin was examined in human chondrocytes (n = 3 for all groups) under basal (control) conditions and after treatment with RNI 249 50 μg/ml (R50), vincaria 10 μg/ml (V10) and their combination (R50 + V10). The * refers to a significant difference from control levels (P < 0.05), and the ** refers to a significant difference from all other groups (P < 0.001), indicating that the combination of R50 and V10 produced significantly greater increases in IGF-1 expression than either V10 or R50 alone.

Direct activation of IGF-1 gene expression was evaluated in primary cultures of human chondrocytes by real time RT-PCR. RNI 249 (50 μg/ml) as well as vincaria (10 μg/ml) when added to the culture media for 48 hr resulted in a significant increase in the expression of IGF-1 (Figure 1), relative to the control, β – actin (P < 0.05). Furthermore, when RNI 249 and vincaria were combined, these effects were additive resulting in a nearly 4-fold increase in IGF-1 gene expression, which was significantly different from RNI 249 or vincaria alone, as well as basal expression (P < 0.001). Cell viability was unaffected by the treatments (data no shown).

**Figure 2:**
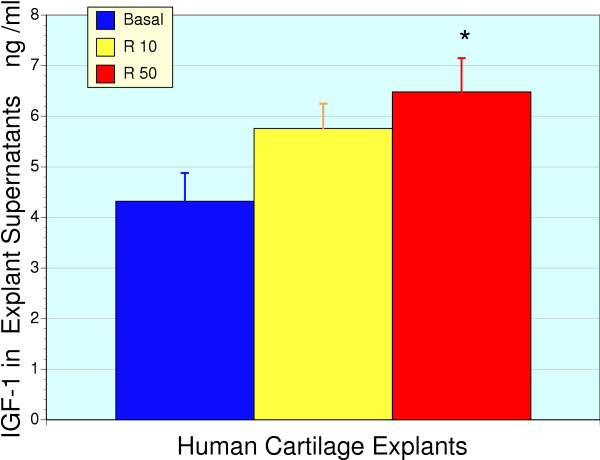
**Production of IGF-1 from human cartilage explants as measured by media IGF-1 levels**. RNI 249 produced a dose-dependent increase in media IGF-1 levels, as determined by ELISA. Only the 50 μg/ml concentration of RNI 249 produced a significant increase over basal, untreated explants (* P < 0.05, n = 6 for all groups).

The potential for RNI 249 to stimulate chondrocyte IGF-1 production was assessed by measurement of IGF-1 in the culture media of cartilage explants. Explants were exposed to RNI 249 at 10 and 50 μg/ml for 48 hours at which time the media was removed for ELISA determination of human IGF-1 levels (Figure 2). RNI 249 treatment resulted in a dose-dependent increase in IGF-1 levels that were significant at the 50 μg/ml dose (P < 0.05). RNI 249 represents the combination of a proprietary *Lepidium meyenii *extract with inert stabilizers as it was noted that the extract alone had a reduced shelf-life, which is corrected in the RNI 249 form. IGF-1 production from human cartilage explants was also enhanced 67% by vincaria alone (10 μg/ml, P < 0.05), consistent with the stimulatory effects of vincaria treatment on IGF-1 gene expression indicated in Figure 1.

### Effects of IL-1β on IGF-1 production

**Figure 3:**
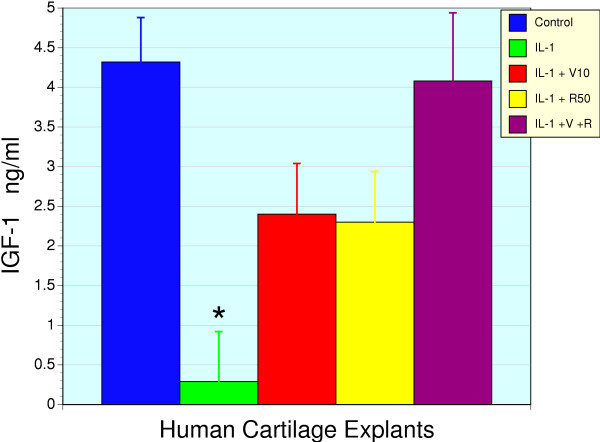
**IGF-1 production from human cartilage explants in response to IL-1β, vincaria and RNI 249 and their combination**. Control, untreated cartilage explants (n = 6) release a defined amount of IGF-1 into the bathing media. However, in IL-1β (5 ng/ml) treated explants IGF-1 levels were immeasurable (n = 6). Co-treatment with either vincaria 10 μg/ml (V10, n=3), or RNI 249 50 μg/ml (R50, n = 6), partially restored IGF-1 production. The combination of vincaria and RNI 249 (IL-1 + V + R, n = 3) produced additive effects that resulted in the restoration of IGF-1 levels to control values despite the presence of IL-1β. The * denotes a significant difference from all other groups (P < 0.01).

While RNI 249 stimulates basal IGF-1 gene expression and production in human cartilage, it was necessary to determine if this action was retained in the presence of IL-1β. Pro-inflammatory cytokines like TNFα and IL-1β are known to silence the expression of the IGF-1 gene and shutdown production in related tissues [31,32,41]. This ability of IL-1β was confirmed in cartilage explants where IL-1β (5 ng/ml) exposed explants displayed a complete suppression of IGF-1 production (Figure 3). Treatment with either vincaria (10 μg/ml) or RNI 249 (50 μg/ml) partially restored IGF-1 production (P < 0.01) but interestingly the combination fully restored IGF-1 levels to baseline, untreated values despite the continued presence of IL-1β.

### IL-1β and GAG release

**Figure 4:**
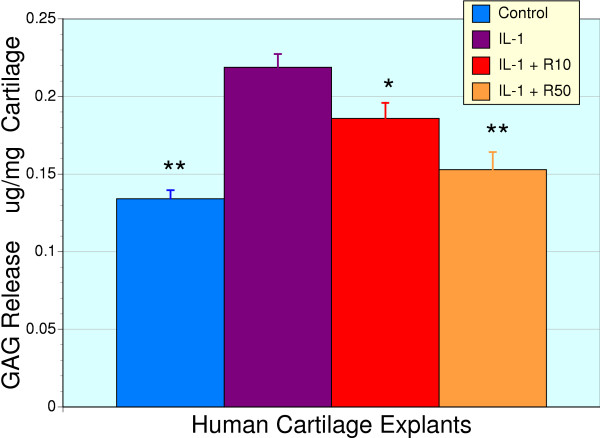
**Normalization of IL-1β enhanced glycosaminoglycan release from human cartilage explants by RNI 249**. Treatment of human cartilage explants (n = 6) with IL-1β (5 ng/ml) results in the release of glycosaminoglycans (GAG) into the media. Administration of RNI 249 produced a dose-dependent decrease of IL-1β induced GAG release that was significant at the 10 μg/ml (R10, * P < 0.05) and 50 μg/ml (** P < 0.01) concentrations. The level of GAG release from control, untreated explants was indistinguishable from those explants treated with IL-1β + RNI 249 (50 μg/ml).

Culture of cartilage explants to study the effects of catabolic mediators on extracellular matrix (ECM) degradation is a well established method. We used this method to study the effects of IL-1β, which induce MMPs and aggrecanases, on human cartilage ECM degradation and its modulation by a natural product extract. Exposure of human cartilage explants to IL-1β resulted in an increase release of glycosaminoglycans into the media (Figure 4, P < 0.05). Co-treatment of RNI 249 and IL-1β resulted in a dose-dependent attenuation of GAG release. Indeed, GAG release from explants that received the 50 μg/ml dose of RNI 249 were not statistically significant from untreated controls (absence of IL-1β) and were significantly improved over the 10 μg/ml dose and IL-1β alone (P < 0.01).

Vincaria (10 μg/ml) also demonstrated an ability to suppress IL-1β induced GAG release (control = 0.141 ± 0.009 μg/mg; IL-1 alone = 0.223 ± 0.010 μg/mg; IL-1β + vincaria = 0.175 ± 0.010 μg/mg: P < 0.01 for IL-1 vs. control or IL-1 + vincaria, n = 3). Potential additive effects associated with the combination of RNI 249 and vincaria was not tested as the individual components provided full protection with media GAG levels were not significantly different from untreated control explants.

### IL-1β and chondrocyte NO production

**Figure 5:**
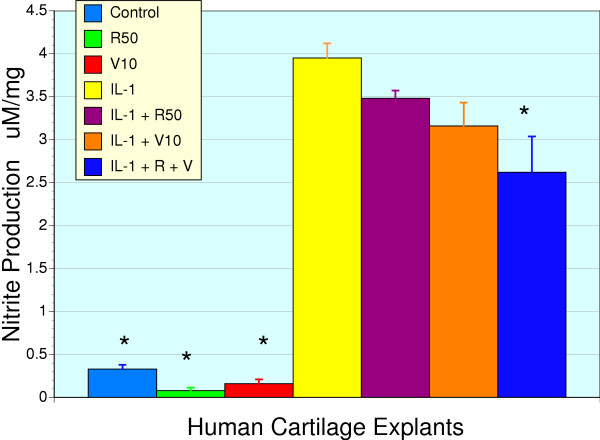
**Effects of vincaria and RNI 249 on basal and IL-1β stimulated nitrite production in human cartilage explants**. Media nitrite levels, a reflection of nitric oxide production, released from human cartilage explants (n = 3) was markedly enhanced by treatment with IL-1β (5 ng/ml, P <0.01). Co-treatment with either vincaria 10 μg/ml (V10) or RNI 249 50 μg/ml (R50) reduced nitrite levels although these changes were not significant. However, the combination of V10 and R50 produced additive effects and a significant reduction in nitrite levels (* P < 0.01 vs. IL-1β). Both V10 and R50 alone, reduced basal nitrite levels when compared to control untreated values but these differences were not significant.

Media nitrite levels in cartilage explants were used as an index of nitric oxide production. Both RNI 249 (50 μg/ml) and vincaria (10 μg/ml) lowered basal nitrite levels (Figure 5) suggesting that there was a mild inherent activation of inducible nitric oxide synthase in these surgical samples. Nevertheless, IL-1β exposure resulted in a marked increase in nitrite levels consistent with an activation of inducible nitric oxide synthase. This response was partially attenuated by vincaria and RNI 249 alone, with additive effects upon co-treatment (P < 0.01). Similar results to these described in cartilage explants were noted in cultured chondrocytes, where RNI 249, vincaria and the combination reduced IL-1β induced nitrite production (p < 0.01) but yet nitrite levels remained significantly above basal nitrite production (data not shown).

## Discussion

Circulating levels of IGF-1 primarily reflect the hepatic production under the influence of growth hormone (GH). While reduced production of GH and IGF-1 are associated with aging and joint dysfunction, poor mobility and increased mortality [36,37,53], the system can be further uncoupled at the level of signal transduction. Both the ability of GH to stimulate IGF-1 formation, and the ability of IGF-1 to activate anabolism can be uncoupled [34-36]. Altered production of IGF binding proteins that limit IGF functionality also contribute to the overall tone of the system [32,43,55-57].

While there are a number of factors including cytokines and poor nutrition, that are thought to contribute to a disruption in IGF-1 functionality [58], the present observations are the first to report that autocrine activation of IGF-1 gene expression in target tissues is a possible solution to these problems. We confirmed in human cartilage explants what has been observed in other tissues that pro-inflammatory cytokines silence IGF-1 production [32,33,36]. In the present case this was achieved with IL-1β, a major determinant of chondrocyte activation and cartilage destruction [3,4,34,59]. However, this is the first report that IGF-1 production can be maintained in the face of these otherwise completely suppressive signals.

The ability of RNI 249, alone and in combination with vincaria, to maintain IGF-1 levels despite the presence of IL-1β has the potential to not only limit cartilage destruction, as was confirmed here with blockade of GAG release, but also to evoke anabolic actions and repair the joint. The present study was not focused on a clear demonstration of an anabolic action. However, there have been numerous studies reporting that exogenous IGF-1 limits joint damage and promotes joint repair [59-64].

It is not known how these botanical extracts achieve these results. We do propose, based on previous observations, that vincaria is effective by limiting the inhibitory actions of IL-1β or the suppressive tone of other pro-inflammatory agents. Vincaria is a remarkably potent redox-based inhibitor of NF-κB and has an IC_50 _for inhibiting LPS-induced TNFα production of 10 ng/ml [25,44,46,47]. We have reported that botanical extracts possessing a similar mechanism of action [21-23] also display chondroprotective actions.

On the other hand, while *Lepidium meyenii *extracts contain catechins [65], they are far weaker antioxidants than vincaria [40,41,59] and as such are unlikely to operate via redox-based actions. *Lepidium meyenii *has primarily been researched for its ability to promote fertility in males and females. The breadth of *Lepidium meyenii*'s actions on fertility is impressive and range from increased fetal growth, reduced miscarriage rates, improvements in conception rates and increased semen volumes and sperm counts [48-52]. It is well documented that *Lepidium meyenii *does not affect the levels of gonadotrophins, sex steroids or prolactin [48,49], and hence the mechanism of action underlying these pro-fertility effects has remained elusive to date. As IGF-1 is a critical determinant of fertility and fetal development we propose that the actions of *Lepidium meyenii *are dictated by a central action of the direct, local activation of IGF-1 production in target tissues, independently of growth hormone. What is of critical interest is that this action can be observed in the presence of IL-1β, a potent inflammatory agent.

RNI 249 alone, as well as vincaria, was able to block the increased release of GAG from cartilage explants by IL-1β. This action of vincaria had been noted previously when combined with a mineral supplement [24], and similar effects have also been observed with potent redox-based anti-inflammatories – green tea epigallocatechin gallate [22] and anthocyanidin enriched pomegranate fruit extract [23]. These redox based natural agents appear to limit cartilage breakdown by blocking the expression, production and release of matrix metalloproteases by activated chondrocytes via a NF-κB and MAPKinase dependent pathway.

It was of interest to note that nitric oxide production, another catabolic pathway in cartilage, was less susceptible to normalization than was GAG release. This follows a pattern seen with other botanical extracts or nutraceuticals, which note reduced but incomplete blockade of IL-1β induced nitric oxide production [22-24]. This suggests that the activation of NO and MMP pathways by IL-1β vary in their responsiveness to these interventions. How this may impacts the clinical response in terms of individual variability and disease targeting remains to be defined. Studer [66] recently described that nitric oxide modifies IGF-1 receptor kinase probably via the formation of nitrosothiols. This structural modification resulted in altered functionality with NO blockade enhancing IGF-1 mediated proteoglycan synthesis.

How RNI 249 limits MMP-mediated events (GAG release) is less clear. A direct suppression of transcription factors like NF-κB is possible, but remote considering its modest antioxidant activity. It is possible that the elevation of IGF-1 production by RNI 249 exerts a negative influence on cartilage catabolism as described with exogenous IGF-1 [54,61-63]. However, as we have not performed studies to demonstrate that RNI 249 actions can be blocked by inhibitors of IGF-1 signal transduction, these events must be considered associations and not causations. It is not clear if autocrine activation of IGF-1 can be blocked by circulating IGF-1 binding proteins and so causative proof-of-principle studies are not simple to execute.

There have numerous and diverse approaches employed to enhance the anabolic actions of IGF-1 ranging from increasing production or independently enhancing functionality. Haupt et al [55] using viral gene transfer studies to synoviocytes corrected articular cartilage degradation in vitro. They observed that by combining IGF-1 with an IL-1 receptor antagonist protein they could both increase proteoglycan production and decrease catabolism via reduced MMP synthesis. They proposed that a combination approach of combining anabolic growth factors with catabolic blockers to prevent matrix degradation would be ideal for healing destructive joint diseases. This is conceptually similar to the approach used in the present study – enhanced autocrine production of IGF-1 and suppression of inhibitory cytokine tone.

Madry et al [64] demonstrated that IGF-1 gene transfer was effective healing for traumatic cartilage repair. Using an in vivo model of cartilage loss in rabbits, Tuncel et al [63] noted that collagen sponges impregnated with recombinant IGF-1 accelerated cartilage restoration. Another approach was described by De Ceuninck et al [55], who noted that disruption of IGF-1 binding to the inhibitory binding proteins via small molecules, resulting in the release of free IGF-1, restores the anabolic response of human chondrocytes. This suggests that a critical regulatory site of IGF-1 bioactivity is the inactivation of circulating IGF-1 by binding proteins. With the appreciation that increased levels of IGF-1 binding proteins have been observed in osteoarthritic cartilage [57] one needs to consider if the optimal approach is to raise circulating IGF-1 levels or alternatively promote local production in an autocrine manner.

While these reports collectively demonstrate that IGF-1 can promote healing and joint restoration and that a multitude of potential approaches to the problem are possible, few are likely to have an immediate impact on health care. By contrast, the combination of the natural products RNI 249 and vincaria has already been commercialized in the USA and Canada under the name "Reparagen" and so it can be considered an immediate and current option in contrast to the elegant but commercially distant approaches of gene transfer or other pharmacological approaches.

A clear limitation of the present study is that it has been performed in vitro, albeit with human cartilage specimens, and therefore it would be appropriate to hold reservations that the present observations may not be translated into the in vivo setting or effective with oral intake. However, we consider that outcomes as being unlikely. In the case of vincaria it has already been shown that it is effective in treating osteoarthritis of the knee in humans with oral administration at the remarkably low dose of only 100 mg/day [47]. Additionally, vincaria has displayed oral bioactivity in animal models of inflammation [25,44]. RNI 249 is also likely to be orally active in terms of enhanced IGF-1 production as it is orally active for anabolic actions in farmed fish (unpublished observations) and *Lepidium meyenii *and related extracts have well defined effects in pregnancy and male fertility upon oral administration [48-51]; effects that are entirely consistent with enhanced IGF-1 production. However, an osteoarthritis orientated clinical trial would address these issues and place the present innovation in an appropriate perspective where one could also address potential benefits to the joint as a whole, including skeletal muscle and bone.

## Conclusion

Joint inflammation and loss of cartilage matrix are not well correlated. Often therapies provide symptomatic relief but fail to restore architecture. The present observations with a novel South American medicinal plant combination that simultaneously addresses catabolic and anabolic activities, suggests that it is possible to directly activate local, chondrocyte IGF-1 production, even in the presence of IL-1β. This action has the potential to provide a therapeutic approach that not only limits joint inflammation and destruction but to promote joint restoration.

## Abbreviations

MMP matrix metalloproteinase

IGF-1 insulin-like growth factor-1

JNK c-Jun N-Terminal kinase

NF-κB nuclear factor kappa B

IL-1β interleukin 1 beta

IL-6 interleukin 6

TNFα tumor necrosis factor alpha

LPS lipopolysaccharide

RT-PCR reverse transcriptase polymerase chain reaction

GAG glycosaminoglycan

NSAID non-steroidal anti-inflammatory drug

COX cyclo-oxygenase

GH growth hormone

IC_50 _inhibitory concentration 50 percent

CIA collagen induced arthritis

## Competing interests

MJSM has a financial interest in Rainforest Nutritionals, Inc

PB has a financial interest in Rainforest Nutritionals, Inc

A SBIR grant award to Rainforest Nutritionals, Inc was used to sponsor the research performed by SA and TMH in this study, and TMH is a scientific advisor to Rainforest Nutritionals, Inc.

## Authors' contributions

MJSM contributed to the design of the study and was the lead author of the manuscript.

SA was the key individual that conducted the studies.

PB contributed to study design and manuscript preparation.

TMH oversaw the performance of the study and contributed to its design and manuscript preparation.

## Pre-publication history

The pre-publication history for this paper can be accessed here:


